# The Role of Trio, a Rho Guanine Nucleotide Exchange Factor, in Glomerular Podocytes

**DOI:** 10.3390/ijms19020479

**Published:** 2018-02-06

**Authors:** Mirela Maier, Cindy Baldwin, Lamine Aoudjit, Tomoko Takano

**Affiliations:** 1 Division of Experimental Medicine, McGill University, Montreal, QC H3A 0G4, Canada; mirela.maier@mail.mcgill.ca; 2 Division of Nephrology, McGill University Health Centre, Montreal, QC H4A 3J1, Canada; cindy.baldwin@mail.mcgill.ca (C.B.); lamine.aoudjit@mcgill.ca (L.A.)

**Keywords:** Trio, CdGAP, Rac1, podocytes, focal segmental glomerulosclerosis, nephrotic syndrome, transforming growth factor β1

## Abstract

Nephrotic syndrome is a kidney disease featured by heavy proteinuria. It is caused by injury to the specialized epithelial cells called “podocytes” within the filtration unit of the kidney, glomerulus. Previous studies showed that hyperactivation of the RhoGTPase, Rac1, in podocytes causes podocyte injury and glomerulosclerosis (accumulation of extracellular matrix in the glomerulus). However, the mechanism by which Rac1 is activated during podocyte injury is unknown. Trio is a guanine nucleotide exchange factor (GEF) known to activate Rac1. By RNA-sequencing, we found that Trio mRNA is abundantly expressed in cultured human podocytes. Trio mRNA was also significantly upregulated in humans with minimal change disease and focal segmental glomerulosclerosis, two representative causes of nephrotic syndrome. Reduced expression of Trio in cultured human podocytes decreased basal Rac1 activity, cell size, attachment to laminin, and motility. Furthermore, while the pro-fibrotic cytokine, transforming growth factor β1 increased Rac1 activity in control cells, it decreases Rac1 activity in cells with reduced Trio expression. This was likely due to simultaneous activation of the Rac1-GTPase activation protein, CdGAP. Thus, Trio is important in the basal functions of podocytes and may also contribute to glomerular pathology, such as sclerosis, via Rac1 activation.

## 1. Introduction

Nephrotic syndrome is characterized by proteinuria (leakage of protein from the blood into the urine), hypoalbuminemia (decreased levels of albumin the blood), and edema. While a variety of diseases can cause nephrotic syndrome, the major sites of injury are the specialized epithelial cells within the filtration unit of the kidney (i.e., glomerulus) called “podocytes”. The podocytes are highly arborized cells and their actin-rich cellular processes (called “foot processes”) interdigitate with those from neighboring podocytes and tightly surround glomerular capillaries. Together with glomerular endothelial cells and the glomerular basement membrane (GBM), podocytes play a critical role in maintaining barrier function of the glomerulus; podocyte injury causes foot processes to retract into the cell body (a phenomenon called foot process effacement), leading to proteinuria. Minimal change disease (MCD) and idiopathic focal segmental glomerulosclerosis (FSGS) are prototypical causes of nephrotic syndrome. Whether FSGS is a distinct disease from MCD or simply a more aggressive form of MCD is still a point of debate [1]. Regardless, in both diseases, unknown humoral factor(s) injure podocytes.

The Rho family of GTPases (RhoGTPases) are well-known regulators of the actin cytoskeleton. RhoGTPases act as a molecular switch, alternating between the active, GTP-bound form, and the inactive, GDP-bound form. They are regulated by three groups of proteins—guanine nucleotide exchange factors (GEFs), GTPase activating proteins (GAPs), and guanine nucleotide dissociation inhibitors (GDIs). GEFs replace GDP for GTP, thereby activating RhoGTPases. GAPs promote hydrolysis of GTP to GDP, thereby rendering them inactive. Finally, GDIs bind the inactive RhoGTPases and keep them sequestered in the cytosol, protected from proteosomal degradation. RhoA, Rac1, and Cdc42 are prototypical RhoGTPases [2].

We and others have shown previously that Rac1 hyperactivity in podocytes contributes to podocyte injury and proteinuria. Transgenic mice with doxycyclin-inducible podocyte-specific expression of constitutively active (CA)-Rac1 rapidly develop proteinuria. Higher transgene expression results in more severe proteinuria and histological changes similar to FSGS [3,4]. Similarly, transgenic zebrafish with low expression of CA-Rac1 specifically in podocytes found in the pronephric glomerulus did not present any abnormalities up to five days post-fertilization. However, increased expression of CA-Rac1 resulted in proteinuria, foot process effacement, and ultimately, progressive edema and death [5]. These results support the notion that Rac1 activity is detrimental to podocytes in a dosage-dependent manner. Rac1 activation in podocytes was also observed in kidney biopsies from patients with FSGS and MCD [3]. Furthermore, mutations in RhoGTPase-regulating proteins lead to increased Rac1 activity and have been found in patients with nephrotic syndrome. Three loss-of-function mutations (ΔD185, R120X, and G173V) in *ARHGDIA* (which encodes GDIα) have been found in patients with congenital or steroid resistant nephrotic syndrome [6,7]. In cultured mouse podocytes, replacing endogenous GDIα with mutant GDIα increased Rac1 activity [8]. The proteinuria and podocyte damage caused by global knockout of GDIα in mice is reversed upon Rac1 inhibition [9]. Similarly, a mutation in *ARHGAP24* (which encodes a Rac1-GAP) was found to be associated with familial FSGS. In mouse podocytes, transfection with this mutant ARHGAP24 elevated Rac1 activity [10].

While the evidence is strong that Rac1 hyperactivity is injurious to podocytes, the mechanism by which Rac1 activity is regulated in podocytes is poorly understood. Thus far, the only Rac1-GEFs found to play a role in podocytes are Vav2 and Vav1. Vav2 was found to activate Rac1 in response to stimulation with Nef, a human immunodeficiency virus, type 1 (HIV-1) accessory protein associated with HIV-1-associated nephropathy (HIVAN), severe proteinuria, and FSGS. In vitro, Nef induces the phosphorylation of Vav2, which in turn activates Rac1 [11]. However, these results await in vivo validation. A recent study used an interleukin-13 (IL-13) overexpression rat model of minimal change-like nephropathy and found that these rats had upregulated expression of Vav1. In vitro, treating human podocytes with IL-13 increases Rac1 activity and induces cytoskeletal reorganization; these changes were abolished by Vav1 knockdown [12]. Thus, Vav1 may play a role in activating Rac1 in podocytes in pathological conditions. Another study investigated the role of two closely related GEFs, Dock1 and Dock5, in podocytes. Although they were expressed in podocytes in vivo, their knockout in the podocyte neither resulted in kidney abnormalities nor protected mice from lipopolysaccharide (LPS)-induced foot process effacement and proteinuria [13]. This suggests that Dock1 and Dock5 do not play an important role in activating Rac1 in podocytes. 

Through gene expression analysis, we identified Trio as a GEF that is highly expressed in podocytes. The current study is aimed at defining the role of Trio in the podocyte’s functions.

## 2. Results

### 2.1. Trio mRNA Is Highly Expressed in Cultured Podocytes and Upregulated in Glomeruli in Patients with FSGS

**Figure 1:**
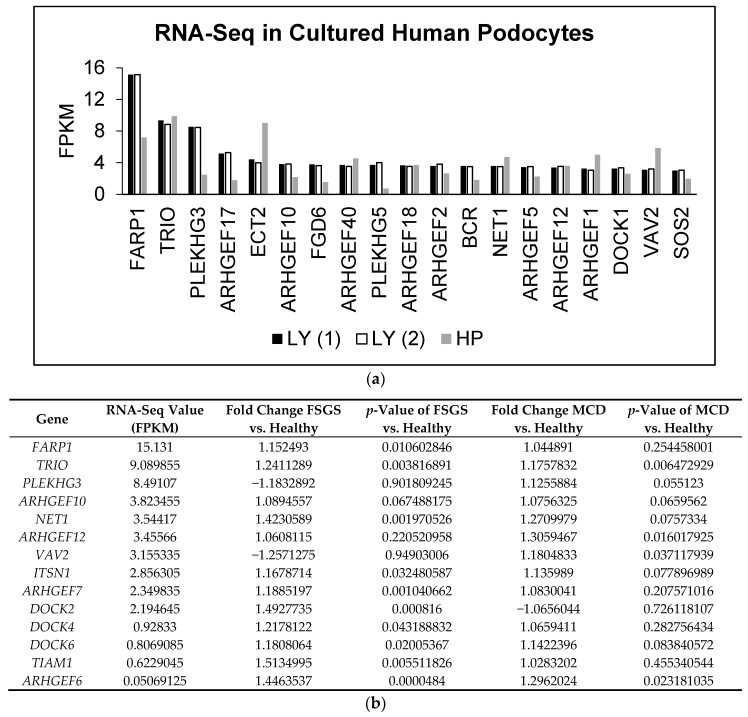

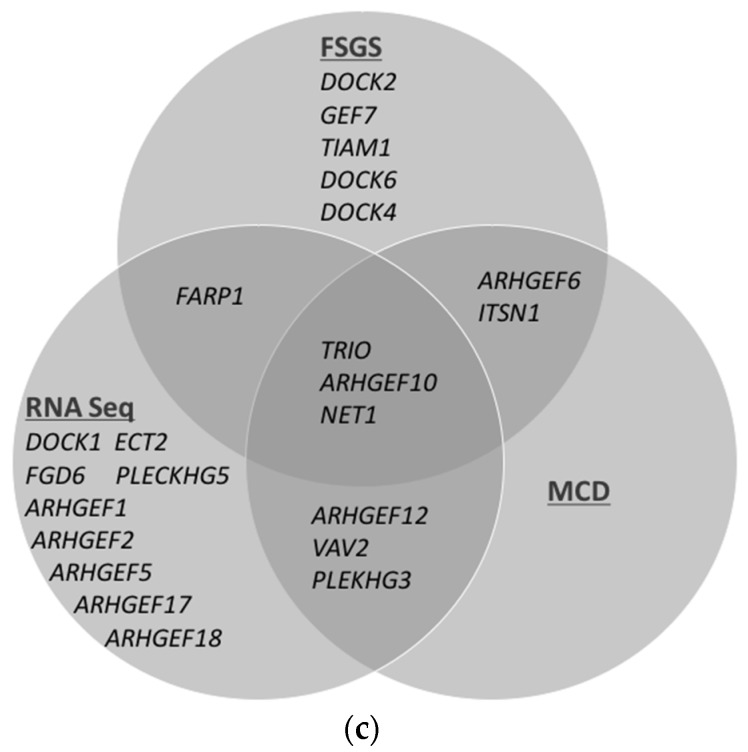
Trio mRNA is highly expressed in cultured podocytes and is upregulated in human proteinuric diseases. (**a**) RNA-seq of two lines of immortalized cultured human podocytes designated as LY (duplicates—labelled (1) and (2)) and HP; (**b**) Nephroseq (Ju podocyte data set) was queried to determine which guanine nucleotide exchange factors (GEFs) are upregulated in focal segmental glomerulosclerosis (FSGS) or minimal change disease (MCD); (**c**) The RNA-seq data and the Nephroseq query results were combined. We identified three candidate GEFs—Trio, Arhgef10, and Net1—that were highly expressed in cultured podocytes and upregulated in both FSGS and MCD, as compared to control.

To date, 83 GEFs have been identified in humans [14]. In order to determine which GEFs are present in podocytes, we performed RNA-sequencing (RNA-seq) on two lines of immortalized cultured human podocytes and cross-referenced the results with the list of GEFs. The mRNA expression levels of GEFs were similar in the two cell lines and the top 19 genes are listed in Figure 1a. We also queried the Nephroseq, an online database that compiles renal gene expression data, and determined which GEFs are upregulated in patients with FSGS or MCD vs. healthy controls. Although many of the GEFs only had a small degree of upregulation in FSGS or MCD, some changes were statistically significant (Figure 1b). Finally, we combined the RNA-seq data with the Nephroseq data and identified three GEFs, Trio, Arhgef10, and Net1, that are highly expressed in cultured human podocytes and significantly upregulated in both MCD and FSGS (Figure 1c). Among the three, Trio was of particular interest; Trio is a dual GEF that activates both Rac1 and RhoA with a preference toward Rac1 [15,16]. It was initially discovered as an interactor of the LAR tyrosine phosphatase, a transmembrane protein found at the focal adhesions [17]. Thus we hypothesized that Trio may modulate focal adhesion dynamics via Rac1 activation, thereby impacting podocyte adhesion to GBM.

### 2.2. Three Isoforms of Trio Are Expressed in Podocytes

**Figure 2:**
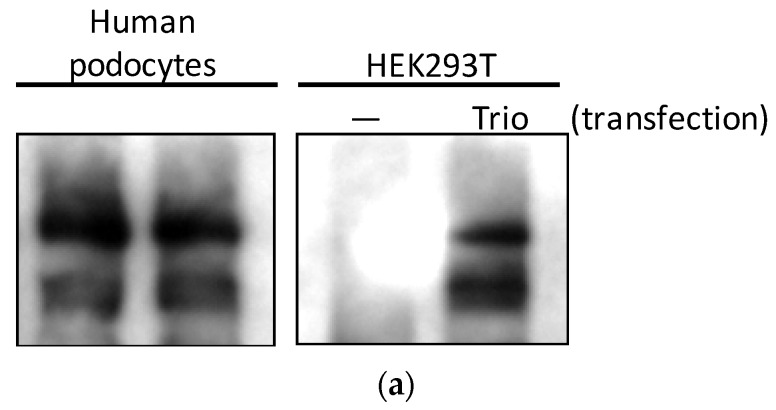

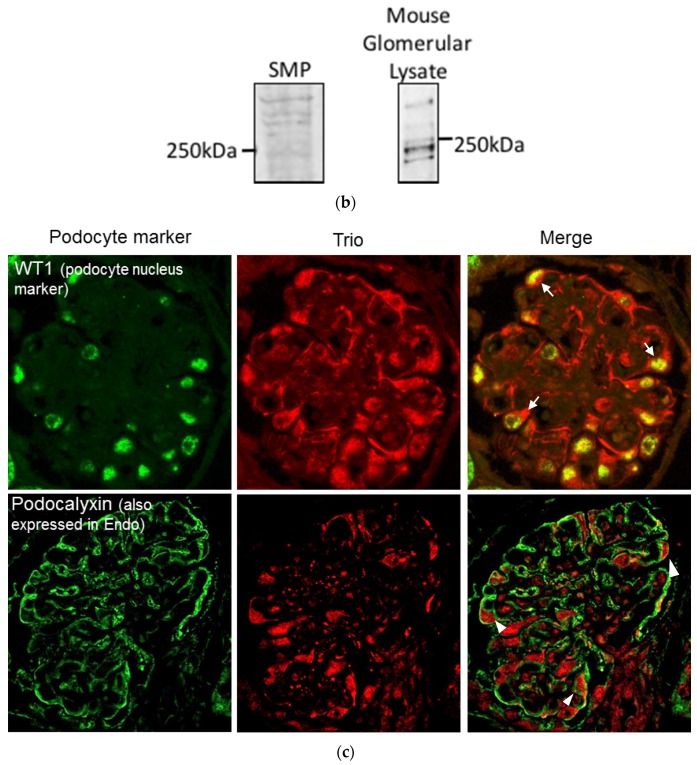
Trio is expressed in podocytes in vitro and in vivo (**a**) HEK293 cells were transfected with full-length Trio or vector alone and lysed one day after transfection, alongside cultured HP cells. Trio antibody detected three isoforms (full-length, D, and A) in HP and transfected HEK293 cells, but showed no signal in untransfected HEK293 cells. Enhanced chemiluminescent (ECL) was used for visualization. (**b**) Immunoblotting revealed Trio expression in cultured mouse podocytes (left) and mouse glomerular lysate (right). LiCor was used for visualization. (**c**) Within the mouse glomerulus, podocyte expression of Trio was shown using immunofluorescence staining. Top: Trio (red) surrounded WT1-positive nuclei (green) with some projections (arrows). Bottom: Trio staining was found between two layers of podocalyxin (green) (arrow heads), which is expressed on the apical membrane of podocyte foot processes. Yellow shows overlapping protein localization. Photos taken at 40× magnification.

First, we studied whether Trio is expressed in podocytes at the protein level. When HEK293 cells were transfected with green fluorescent protein (GFP)-tagged full-length Trio, immunoblotting of the lysates showed three bands corresponding to the known three isoforms; full-length (334 kDa), isoform D (303 kDa), and isoform A (254 kDa) (Figure 2a) [18]. These three bands were also observed in immortalized human podocytes (Figure 2a). Multiple isoforms of Trio were also found in immortalized mouse podocytes (MP) and glomeruli isolated from mice (Figure 2b). Trio protein expression in podocytes in vivo was confirmed using immunofluorescence staining on mouse glomeruli. Trio staining was found surrounding nuclei positive for Wilms’ tumor-1 (WT1), a podocyte-specific nuclear transcription factor in the mature glomerulus; some of the staining was observed in projections extending from the nucleus. Furthermore, Trio staining along the capillary loop was found between two layers of podocalyxin staining, a protein found at the apical side of podocyte foot processes and in endothelial cells (Figure 2c), likely suggesting the presence of Trio in the foot processes. These staining patterns suggest that the Trio is expressed in the podocyte cell body, in primary processes, and in foot processes.

### 2.3. Trio Contributes to Basal Rac1 Activity and Cell Size

**Figure 3:**
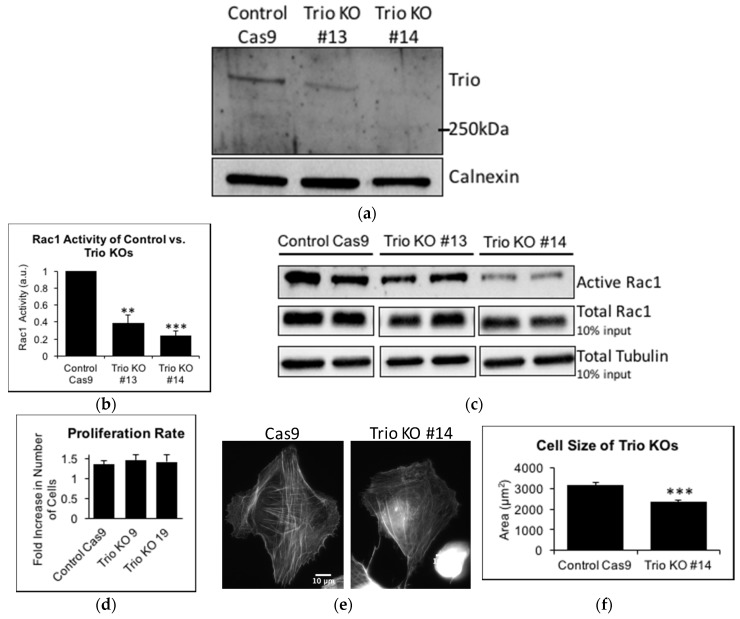

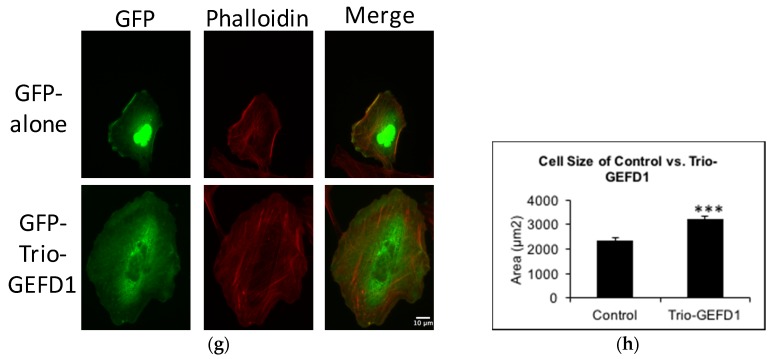
Trio Contributes to Basal Rac1 Activity and Cell Size (**a**) The CRISPR/Cas9 system was used to create Trio KO human podocytes. Monoclonal populations were selected and tested for Trio expression using immunoblotting. Although the KO was not complete, it decreased Trio expression by 60% (Trio KO #13) or 58% (Trio KO #14) when normalized to calnexin and then to control Cas9. ECL was used for visualization; (**b**,**c**) Cdc42/Rac interactive binding (CRIB) pulldown (PD) assays revealed that both Trio KO #13 (0.39 ± 0.10, *n* = 3, ** *p* < 0.01 compared to control Cas9) and Trio KO #14 (0.24 ± 0.06, *n* = 3, *** *p* < 0.001 vs. control Cas9) had decreased Rac1 activity compared to control Cas9. ECL was used for visualization and quantification was done using densitometric analysis. Results were normalized to tubulin and then to control Cas9 cells; (**d**) MTT assay showed that the proliferation rate of Trio KOs did not differ from that of control Cas9 cells between 24 h and 48 h after plating (*y*-axis represents the value at 48 h over the value at 24 h). Trio KOs #9 and #19 also had decreased Trio expression (see Figure S1) and were used for the MTT assay; (**e**,**f**) Control Cas9 and Trio KOs were fixed and stained for phalloidin. Trio KOs (2349 ± 114 μm^2^, *n* = 89 cells from two experiments, *** *p* < 0.001 vs. control Cas9) were significantly smaller than control Cas9 (3167 ± 140 μm^2^, *n* = 81 from two experiments). Scale bar in Cas9 photo (**e**) is 10 μm and is the same for Trio KO photo (**g**,**h**) GFP-alone or GFP-Trio-GEFD1 were expressed in HP and cells were stained for phalloidin. GFP-Trio-GEFD1 expressing cells were larger (3223 ± 131 μm^2^, *n* = 65 from two experiments, *** *p* < 0.001 vs. GFP-alone) than GFP-alone expressing cells (2327 ± 142 μm^2^, *n* = 61 from two experiments). Scale bar in (**g**) is 10 μm.

Next, we studied whether Trio plays a role in controlling Rac1 activity in podocytes. To do this, we applied the CRISPR/Cas9 system in human podocytes, expecting to completely knock out Trio expression. The resulting cells showed greatly reduced expression of Trio; however, there was some residual expression (Figure 3a, see Methods for possible explanation). Nevertheless, these cells were designated as Trio KO. As control, Cas9 cells (control Cas9) without the sgRNA were established. When Rac1 activity was studied using a Cdc42/Rac Interactive Binding (CRIB) pulldown (PD) assay under basal, non-stimulated conditions, Trio KO cells showed significantly lower Rac1 activity, as compared with control Cas9 cells (Figure 3b,c). 

An increase in Rac1 activity is generally associated with increased cell size; we showed previously that transfection of mouse podocytes with CA-Rac1 makes cells larger [19]. We quantified the size of Trio KO cells, by using phalloidin staining of the actin cytoskeleton. Trio KOs were significantly smaller, compared to control Cas9 (Figure 3e,f). Conversely, we generated HP cells expressing a GFP-tagged constitutively active form of Trio, which only contains the Rac1 GEF domain (called the Trio GEFD1 domain), without the inhibitory spectrin-like repeats or the RhoA GEF domain (the GEFD2 domain). Compared to HP expressing GFP-alone, the HP with GFP-Trio-GEFD1 were significantly larger (Figure 3g,h). 

Trio knockdown in cultured glioblastoma cells was shown to decrease proliferation [20]. However, when we compared cell proliferation between Trio KOs and control Cas9 using a 3-(4,5-dimethylthiazol-2-yl)-2,5-diphenyltetrazolium bromide (MTT) assay, we did not find any difference in the time frame studied (between 24 and 48 h after plating). It is possible that no difference was found due to the incomplete nature of the Trio KO. Together, the results indicate that in podocytes, Trio plays a role in activating Rac1 and increasing cell size through the GEFD1 domain, but may not affect cell proliferation.

### 2.4. Trio Affects Motility, Attachment, and Vinculin Distribution of Podocytes

**Figure 4:**
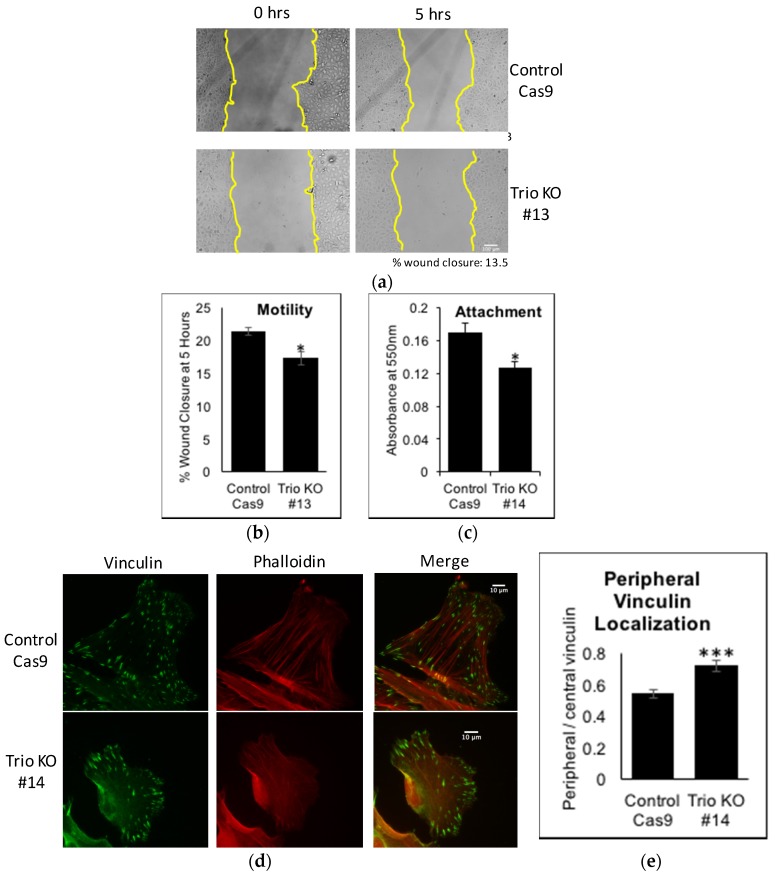
Trio affects motility, attachment, and vinculin distribution. (**a**) Control Cas9 and Trio KOs were allowed to grow to confluency, serum starved overnight, and then a scratch wound was created. Images were taken at time 0 and 5 h after; (**b**) Cell motility was calculated from percent wound closure from 0 to 5 h. Control Cas9 cells had a greater wound closure than Trio KOs (control Cas9: 21.4 ± 0.55, Trio KOs: 17.3 ± 0.99, *n* = 4, * *p* < 0.05 vs. control Cas9). The yellow lines have been drawn to easily visualize the two sides of the wound. Scale bar in (**a**) is 100 μm; (**c**) The same number of control Cas9 and Trio KOs were plated on laminin 521 and allowed to attach for 1.25 h. Attached cells were quantified by crystal violet as described in Methods. Control Cas9 cells had more attachment than Trio KOs (absorbance values at 550 nm: control Cas9: 0.17 ± 0.01, Trio KO: 0.13 ± 0.01, *n* = 3–4, * *p* < 0.05 vs. control Cas9); (**d**) Control Cas9 and Trio KOs were plated overnight on laminin 521, fixed, and stained for phalloidin (red) and vinculin (green). Co-localization is shown in yellow in merged photo. Scale bar in (**d**) is 10 μm. The amount of vinculin at the periphery was quantified; (**e**) Quantification of peripheral vs. central vinculin staining from (**e**) was performed as described in Methods. Trio KOs had more peripheral vinculin than control Cas9 (control Cas9: 0.55 ± 0.02, *n* = 21 cells counted from two experiments; Trio KO: 0.72 ± 0.04, *n* = 19 cells counted from two experiments, *** *p* < 0.001 vs. control Cas9).

Active Rac1 is generally found at the leading edge of a moving cell, where it promotes actin remodeling and focal complex turnover, thereby increasing motility [21]. Trio knockdown in glioblastoma cells [20] or HeLa cells [16] decreased migration. This led us to explore Trio’s role in migration in podocytes. Wound healing assay revealed that Trio KOs have a significant migration impairment, with decreased wound closure by five hours (Figure 4a,b).

For podocytes to properly function as a filtration barrier, it is essential that podocytes are securely attached to the GBM. Thus, we next studied the role of Trio in podocyte attachment to laminin 521, a major extracellular matrix component of the mature GBM. When human podocytes were trypsinized and plated on the surface coated with laminin 521, Trio KOs showed significantly decreased attachment, as compared with control Cas9 cells (Figure 4c). When focal adhesion complexes were visualized by staining for vinculin (a component of focal adhesion complex), control Cas9 cells had focal adhesion complexes spread throughout the cell. In contrast, in Trio KOs, focal adhesion complexes were localized more at the periphery of the cell and sparse in the central part of the cell where focal adhesion complexes are expected to be more mature and stable [22] (Figure 4d,e). Thus, Trio may contribute to secure attachment of podocytes to GBM/extracellular matrix by stabilizing focal adhesion complexes at the basal aspect of the cell.

### 2.5. TGFβ1 Increases Trio and Rac1 Activity in Podocytes

**Figure 5:**
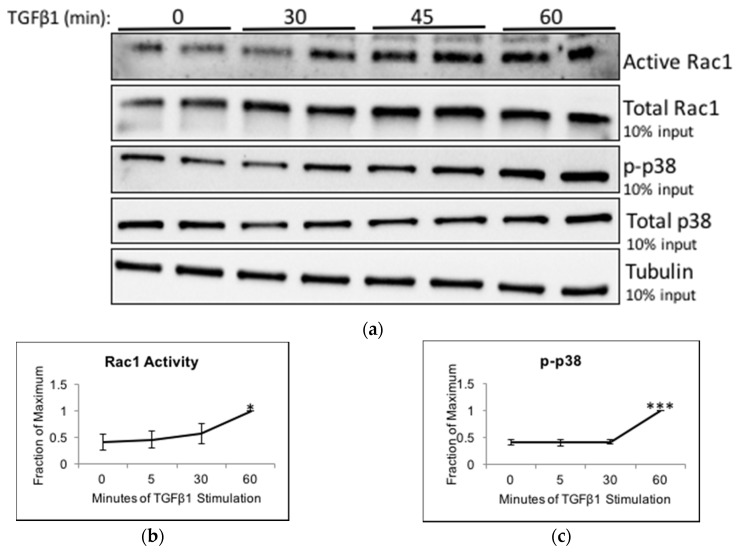

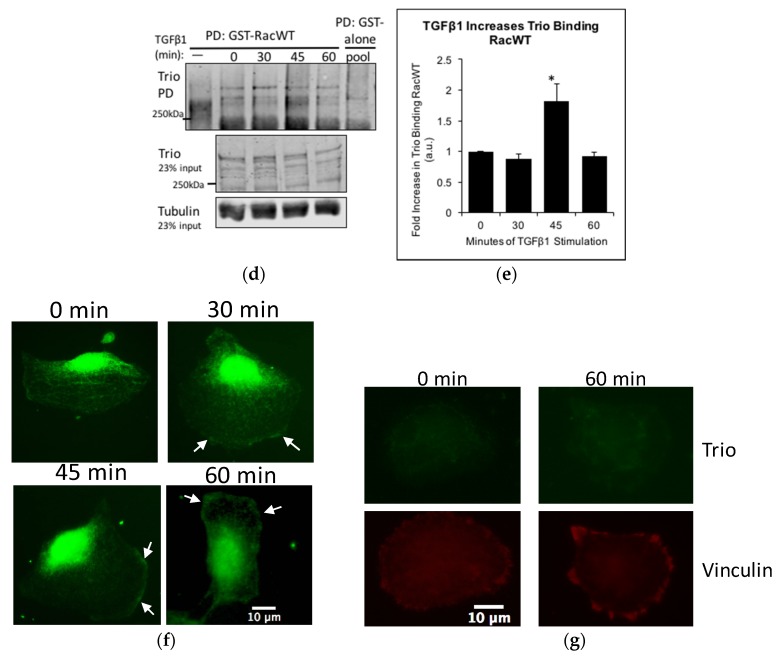
TGFβ1 increases Rac1 activity and Trio binding to Rac1. (**a**–**c**) HP cells were stimulated with TGFβ1 (10 ng/mL) for the indicated times and cells lysates were subjected to CRIB PD. Precipitates and total lysates were immunoblotted for Rac1, p-p38, and p38. Experiment was done in duplicates and visualized using ECL; (**b**,**c**) Densitometric analysis. Results were normalized to tubulin and then to the highest Rac1 activity or p-p38 level (found at 60 min) (*n* = 3–4, * *p* < 0.05 and *** *p* < 0.001 compared to 0 min); (**d**) HP cells were stimulated with TGFβ1 as in (**a**) and cell lysates were subjected to PD using GST-Rac1. The PD samples and total lysates were immunoblotted for Trio and tubulin. Two negative controls were used: (i) the GST-Rac1 beads were alone (left lane labelled) and (ii) a mixture of the lysates were pulled down with GST-alone (rightmost lane). No Trio signal was found in either of these lanes, suggesting that the PD and antibody were specific. LiCor was used for visualization; (**e**) Densitometric analysis. Results were normalized to tubulin and then to 0 min. At 45 min, Trio binding to GST-Rac1 increased 1.82-fold ± 0.29, *n* = 3–4, * *p* < 0.05 vs. unstimulated; (**f**,**g**) Control (**f**) HP or Trio KOs (**g**) were plated overnight on laminin 521 and then stimulated with TGFβ1 (10 ng/mL). Cells were fixed and stained for Trio. In control cells, TGFβ1 increased Trio staining at the cell membrane of the lamaellipodia (arrows). Very low Trio signal was detected in Trio KOs, and TGFβ1 did not affect Trio localization in KOs. Vinculin staining (red) is shown to demonstrate the position of the cell. Vinculin staining intensity was similar between the control (not shown) and Trio KO cells. Scale bar in (**f,g**) is 10 μm.

We next sought to find an extracellular ligand that could modulate the activity of Trio towards Rac1. The first step was to find an activator of Rac1. We chose to investigate transforming growth factor β1 (TGFβ1), a pro-fibrotic cytokine that has been implicated in the pathogenesis of FSGS and is highly expressed in podocytes from patients with idiopathic FSGS [23,24]. Furthermore, TGFβ1 has been shown to activate Rac1 in glomerular mesangial cells [25]. Human podocytes were stimulated with TGFβ1 for up to 60 min, and lysates were subjected to CRIB PD. Rac1 activity was increased at 60 min of stimulation, but not at 30 or 45 min (Figure 5a,b). We also studied p38 mitogen active protein kinase (MAPK), which is activated partially by Rac1 in response to LPS and is a marker of podocyte injury [26]. By immunoblot analysis, we observed that at 60 min, TGFβ1 increases phosphorylation of p38 (p-p38) on Thr180/Tyr182, which represents its active form (Figure 5a,c).

Next, we investigated whether Trio contributed to the TGFβ1-induced Rac1 activation. In 2006, Garcia-Mata et al. [27] suggested that a nucleotide-free mutant of Rac1 (RacG15A) would bind GEFs with high affinity and used pulldown with Glutathione *S*-transferase (GST)-Rac1G15A as a method of measuring GEF activity [27]. However, to our knowledge, there are no studies comparing the affinity of GEFs to Rac1G15A versus wild-type Rac1. We found that that Trio is unable to bind GST-Rac1G15A; however, it can bind GST-Rac1 (Figure S2). Thus, we used PD with GST-Rac1 to study the affinity of Trio to Rac1, which likely reflects Trio’s activity. In human podocytes, a time course of TGFβ1 showed that Trio is pulled down most by GST-Rac1 after 45 min of stimulation (Figure 5d,e). Immunostaining of human podocytes revealed that intracellular localization of Trio is also altered by TGFβ1. In unstimulated cells, Trio staining was diffuse throughout the cytosol and was particularly intense around the nucleus. Within 30 min of TGFβ1 stimulation, Trio staining at the cell membrane of the lamellipodia was clearly observed (Figure 4f), where Rac1 activation is known to occur when cells are moving [21]. The results suggest that TGFβ1 mobilizes Trio at the leading edge of the cells and increases the affinity of Trio to Rac1, both leading to subsequent activation of Rac1 and cell motility. 

**Figure 6:**
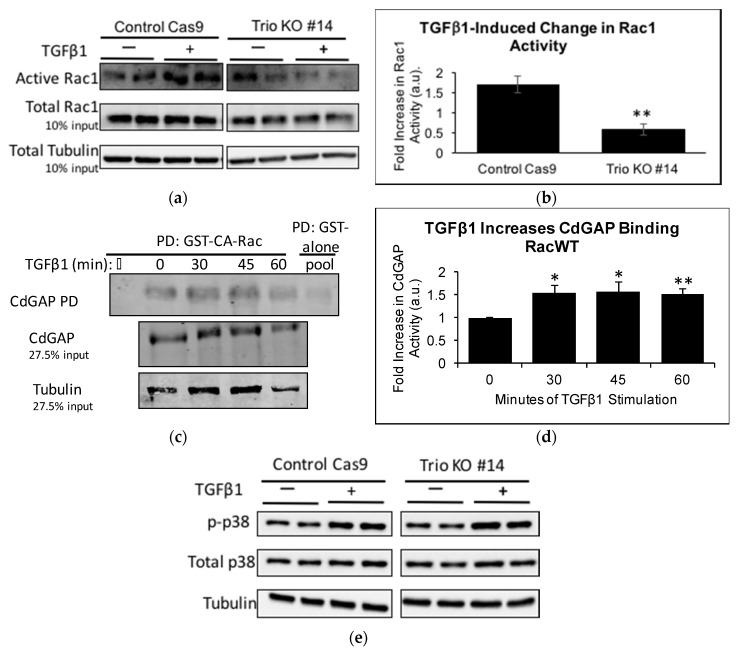

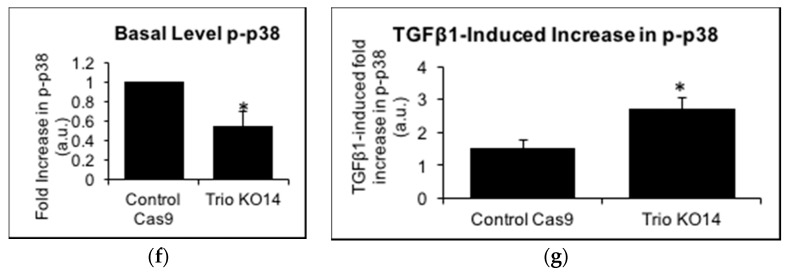
TGFβ1 decreases Rac1 activity in Trio KOs, while increasing CdGAP activation in control cells. (**a**) Control Cas9 and Trio KOs were incubated with TGFβ1 (10 ng/mL) for 60 min. Cell lysates were subjected to CRIB PD and immunoblotting for Rac1 and tubulin. ECL was used for visualization; (**b**) Densitometric analysis. Results were first normalized to tubulin and then fold increase of +TGFβ1 over −TGFβ1 was calculated. In control Cas9 cells, TGFβ1 increased Rac1 activity 1.72-fold ± 0.20, *n* = 4; whereas in Trio KOs, TGFβ1 decreased Rac1 activity 0.59-fold ± 0.13, *n* = 4. ** *p* < 0.01 vs. control Cas9; (**c**) HP were treated with TGFβ1 for the indicated times and cell lysates were pulled down with GST-CA-Rac1. Precipitates and lysates were immunoblotted for CdGAP and tubulin and visualized using LiCor; (**d**) Densitometric analysis. Levels of active CdGAP were normalized to CdGAP levels in the total lysate (i.e., input) and then to 0 min. CdGAP activity was increased at 30 min (1.55-fold ± 0.16, * *p* < 0.05 vs. unstimulated), 45 min (1.56-fold ± 0.21, * *p* < 0.01 vs. unstimulated), and 60 min (1.53-fold ± 0.12, ** *p* < 0.05 vs. unstimulated), *n* = 3–4; (**e**) Control Cas9 cells and Trio KOs were stimulated with TGFβ1 (10 ng/mL, 60 min), cell lysates were immunoblotted for p-p38 and total p38, and protein was visualized using ECL; (**f**) Densitometric analysis. Basal (unstimulated) levels of p-p38 were normalized to tubulin and then to control Cas9 levels. Trio KOs had 0.55 ± 0.28 fold decrease, *n* = 4, * *p* < 0.05 versus control Cas9; (**g**) Results were normalized to tubulin and then fold increase of +TGFβ1 over −TGFβ1 was calculated. TGFβ1 increased p-p38 more in Trio KOs than in control Cas9 (control Cas9: 1.54 ± 0.45 fold, Trio KO: 2.71 ± 0.70 fold, *n* = 4, * *p* < 0.05 vs. control Cas9).

To ascertain that Trio does indeed contribute to TGFβ1-induced Rac1 activation, CRIB PD was performed on control Cas9 and Trio KOs. TGFβ1 increased Rac1 activity in the control Cas9 as expected; however, to our surprise, Rac1 activity decreased significantly in Trio KOs upon TGFβ1 stimulation at 60 min (Figure 6a,b). This led us to hypothesize that TGFβ1 may be simultaneously activating a Rac1-GAP. In mammary tumor explants expressing activated Neu/ErbB-2 receptor, the Rac1/Cdc42-GAP, CdGAP, was required for TGFβ1 to induce migration and invasion [28]. Furthermore, by re-analyzing the RNA-seq data, we found that ARHGAP31 (encoding CdGAP) mRNA is expressed in both LY and HP cells (Fragments Per Kilobase Million (FPKM) values in LY cells (duplicates): 1.67, 1.74; FPKM values in HP cells: 2.53) (data not shown). Thus, we studied if CdGAP is activated by TGFβ1 in podocytes. We used an assay that specifically pulls down active Rac1-GAPs, which consists of GST-tagged CA-Rac1 (GST-CA-Rac) [27], followed by immunoblotting for CdGAP. A time course of TGFβ1 treatment showed that CdGAP binding to CA-Rac1 was significantly increased at 30, 45, and 60 min (Figure 6c,d). The results suggest that TGFβ1 induces both Trio and CdGAP activation, which have opposing effects on Rac1 activity. We speculate that in control HP, Trio wins the tug-of-war and leads to increased Rac1 activation in response to TGFβ1, while in Trio KO cells, Rac1 deactivation by CdGAP becomes apparent. Precise interplay among numerous GEFs and GAPs expressed in HP requires further investigation.

Finally, we also studied p38 activation. Compared to control Cas9, Trio KOs have decreased basal levels of p-p38 (Figure 6e,f), consistent with the decreased basal levels of Rac1 activity in these cells. Considering that TGFβ1 decreases Rac1 activity in Trio KOs, and p38 activation occurs partially through Rac1, we anticipated that TGFβ1 would not activate p38 in Trio KOs. However, to our surprise, we found that TGFβ1 induces a greater activation of p38 in Trio KOs than in control Cas9 (Figure 5e,g). This suggests that Rac1 is a minor activator of p38 downstream of TGFβ1 in podocytes.

## 3. Discussion

We have identified Trio as a Rac1 activator in podocytes. Trio was among the most highly expressed GEF at the mRNA level in cultured human podocytes and its glomerular mRNA levels were significantly upregulated in MCD and FSGS in humans (Figure 1). It was expressed at the protein level in cultured human and mouse podocytes and podocytes in vivo in the mouse kidney (Figure 2). In human podocytes, three isoforms of Trio (full-length, A, and D) were expressed, all of which contain the GEFD1 domain responsible for Rac1 activation (Figure 2). This is novel, as expression of the D and A isoforms was previously thought to be specific to the nervous system [18]. Reduced Trio levels in human podocytes resulted in decreased Rac1 activity, cell size, and motility, while expression the GEFD1 domain of Trio increased cell size (Figure 3). Reduced Trio levels also led to redistribution of focal adhesion complexes and decreased ability to attach to the extracellular matrix (Figure 4). When podocytes were stimulated with the pro-fibrotic cytokine, TGFβ1, Rac1 was activated, which was in part dependent on Trio (Figure 5). When the Trio levels were reduced, TGFβ1 induced reduction of Rac1 activity, likely mediated by activation of the Rac1-GAP, CdGAP (Figure 6). 

Phenotypical changes of cultured podocytes upon reduced Trio expression, such as reduced cell size, motility, and adhesion to matrix, along with decreased Rac1 activity, are highly suggestive that Trio plays important roles in the basal morphology and function of podocytes. We showed previously that Rac1 activity is high in immature podocytes but gradually decreases as podocytes mature; in fully developed glomeruli, active Rac1 was barely detectable in podocytes [3,29]. Since cultured podocytes may retain some characteristics of immature podocytes (e.g., lack of foot processes), we speculate that Trio may be an important Rac1 activator when podocytes are going through earlier development.

On the other hand, Trio contributed to Rac1 activation induced by the pro-fibrotic cytokine, TGFβ1. TGFβ1 has been shown to play important roles in podocyte apoptosis/loss and development of glomerulosclerosis [30,31,32,33,34]. We and others showed that Rac1 hyperactivation in podocytes induces podocyte foot process effacement and, if sustained, podocyte loss and glomerulosclerosis [3,4]. It is conceivable that in the context of TGFβ1 overproduction in the glomerulus, e.g., diabetic nephropathy [35,36,37], TGFβ1 activates Rac1 in podocytes, ultimately leading to podocyte loss and glomerulosclerosis. Interestingly, TGFβ1 simultaneously promotes Trio and CdGAP activation; in the presence of Trio, Rac1 is activated by Trio more than it is deactivated by CdGAP. When Trio expression is reduced, TGFβ1 decreases Rac1 activity. This is curious, considering that there are numerous other GEFs that can possible compensate for the lack of Trio; it suggests a dominant role of Trio as a Rac1-GEF in podocytes. Alternatively, Trio may act as a scaffold/adopter protein and may be necessary for other GEFs to activate Rac1. Consistent with this possibility, several studies demonstrated that Trio knockout or knockdown in HeLa cells inhibits Rac1 activation, cell migration and spreading in the presence of many other Rac1-GEFs [16]. In either case, Trio could be a potential therapeutic target in sclerotic glomerular disease.

The mechanism by which TGFβ1 activates Trio remains unknown. Trio’s ability to activate Rac1 has been shown to be regulated by a number of proteins, including Fyn (a Src family tyrosine kinase) [38], disrupted-in-schizophrenia 1 (DISC1) [39], Kidins220/ARMS (an integral membrane protein) [40], Trio-associated repeat on actin (Tara) (an F-actin binding protein) [41,42], and Hsc70 (a constitutively and ubiquitously expressed ATP-dependent chaperone) [43]. All of these proteins were expressed in immortalized human podocytes at the mRNA level, as seen by the RNA-seq (Table S1). In Nephroseq, Hsc70 is increased 1.15-fold (*p* = 0.002) in FSGS vs. healthy living donor (in the Ju CKD dataset). Interestingly, TGFβ1 has been previously reported to increase Hsc70 expression in cultured chicken embryo cells [44]. Although we did not find Hsc70 expression to be increased within 60 min of TGFβ1 stimulation [45], TGFβ1 may increase Hsc70’s interaction with Trio, thereby activating Trio’s Rac1-GEF activity. Further studies will be required to determine the mechanisms of regulation of Trio activity in podocytes.

In conclusion, we found that Trio plays a role in activating Rac1 in podocytes both in basal conditions and when stimulated with TGFβ1. We speculate that Trio may have dual roles; it may play important roles in podocyte development but it may also regulate pathological Rac1 hyperactivation in the context of TGFβ1-mediated podocyte injury and glomerulosclerosis. Elucidation of the precise role of Trio in podocyte development and glomerulosclerosis will require further studies including conditional and inducible Trio knockout in vivo. 

## 4. Materials and Methods

### 4.1. Materials

Tissue culture media, blasticidin, and puromycin were from Wisent (Saint-Bruno, QC, Canada). Interferon-γ and laminin 521 were from Cedarlane (Burlington, ON, Canada). TGFβ1 was from Peprotech (Dollard des Ormeaux, QC, Canada). Protein assay dye reagent concentrate and electrophoresis reagents were from BioRad Laboratories (Mississauga, ON, Canada). Normal goat serum and lipofectamine 2000, were from Invitrogen (Burlington, ON, Canada). Aqua Mount and enhanced chemiluminescent (ECL) were from Thermo Scientific. Glutathione-Sepharose 4B beads were from GE Healthcare (Baie-D’Urfe, QC, Canada). Complete Mini Protease Inhibitor Cocktail was from Roche Diagnostics (Montreal, QC, Canada). The Nucleofector Kit for Basic Mammalian Epithelial Cells was from Lonza (Mississauga, ON, Canada). 

pGEX2t-GST-CA-Rac1, pGEX2t-GST-RacWT and pGEX2t-GST-alone were from generated by subcloning [46] CA-Rac1 and Rac1WT into pGEX2t from GE Healthcare Life Science (Chicago, IL, USA). The pLenti CMV rtTA3 Blast (w756-1) was a gift from Eric Campeau (Addgene plasmid # 26429, Addgene, Cambridge, MA, USA). pMD2.G (Addgene plasmid # 12259) and psPAX2 (Addgene plasmid # 12260) were gifts from Didier Trono. The lentiviral Cas9 and sgRNA Trio were from Genecopoeia (Thorold, ON, Canada). The pTRE2-GFP-alone and gTRE2-GFP-Trio-GEFD1 were kind gifts from Dr. Nathalie Lamarche-Vane (McGill University) [43].

Rhodamine-conjugated phalloidin was from PromoKine (Heidelberg, Germany). Rabbit anti-p-p38, anti-rabbit IgG Alexa Fluor 488 and anti-mouse IgG Alexa Flour 555 were from Cell Signalling (Beverly, MA, USA). Mouse anti-α-tubulin, mouse anti-synaptopodin, rabbit anti-p38, Horseradish peroxidase (HRP)-conjugated streptavidin, HRP-conjugated anti-rabbit and anti-mouse antibodies were from Abcam (Cambridge, MA, USA). Rabbit anti-Trio antibodies were from Cedarlane (Burlington, ON, Canada) or Santa Cruz (Dallas, TX, USA). Mouse anti-Rac1 was from Millipore (Etobicoke, ON, Canada). Mouse anti-Hsc70, mouse anti-WT-1, mouse anti-myc were from Santa Cruz. Rabbit anti-calnexin was from Stressgen (San Diego, CA, USA). IRDye LiCor secondary antibodies were from LiCor (Burlington, ON, Canada). 

### 4.2. Cell Culture and Transfection

Two lines of conditionally immortalized human podocytes (designated as HP and LY8H3) were kind gifts from Dr. Moin Saleem (University of Bristol) and used as published previously [47]. Briefly, cells were cultured in RPMI 1640, 10% fetal bovine serum (FBS), 1% penicillin/streptomycin (P/S), maintained at 33 °C and differentiated at 37 °C for 1 week for experiments. Trio KOs derived from HP did not tolerate differentiation thus were used at 33 °C. For all experiments with human podocytes, HP line was used, except LY cells were also used for RNA-sequencing. Conditionally immortalized mouse podocyte were obtained from Dr. Stuart Shankland (University of Washington), and cultured in DMEM, 10% FBS, and 1% P/S and interferon γ (10 U/mL), as previously described [48]. HEK293 were cultured in DMEM, 10% FBS, and 1% P/S. 

### 4.3. Generation of Trio KOs and Nucleofection

For lentivirus packaging, HEK293 were transiently transfected with lentiviral Cas9, psPAX2 (packaging plasmid), pMDG.2 (envelope plasmid) at a ratio of 4:3:1 with Lipofectamine 2000 transfection reagent. Cells were incubated for 18 h, after which the medium was replaced. Twenty-four and forty-eight hours after replacing medium, the virus-containing medium was collected and filtered through a 0.45 μm filter. HP cells were incubated with 1 mL of this lentiviral particle-containing medium for 24 h. Cells were then selected with blasticidin (8 μg/mL) to create the control Cas9 HP. The process was then repeated on the control Cas9 cells using sgRNA targeting Trio to create Trio KOs, which were selected for by blasticidin and puromycin (0.2 μg/mL). Trio KOs underwent a limiting dilution to create monoclonal cell lines. While Trio expression was greatly reduced, it was not completely abolished. This is likely because HP have greatly reduced viability when not surrounded by other cells. Thus, despite the limiting dilution, it is likely that the cell lines created were not purely monoclonal, but rather grew from a small colony of cells, which may have been a mixture of knockout and non-knockout cells.

HP rtTA3 cells were created by retroviral transfection of HP cells with pLenti CMV rtTA3 (see Materials, Section 4.1) and selected for using blasticidin. Nucleofection of these cells was done as described before [19]. Briefly, 50 × 10^4^ HP rtTA3 cells were mixed with 100 μL of Nucleofector Solution mix (as per instructions from Amaxa Basic Nucleofector Kit Primary Mammalian Epithelial Cells). This mixture was then added to 1 μg of DNA (pTRE2-GFP-Trio-GEFD1 or pTRE2-GFP alone), nucleofected (using the Amaxa Biosystems Nucleofector I, program S-05), and added to laminin 521-covered coverslips. After 8 h, doxycycline (1 μg/mL) was added overnight to induce expression.

### 4.4. RNA-Sequencing

Two human podocyte lines (LY and HP) were differentiated by a temperature switch to 37 °C for 1 week and RNA was prepared using RNeasy mini kit (QIAGEN). RNA-seq was performed at the McGill University and Genome Quebec Innovation Centre for Eukaryotic protein coding transcripts.

### 4.5. Nephromine/Nephroseq Analysis

Our original search for candidate GEF was conducted on Nephromine using the “Ju Podocyte” dataset, which consisted of 436 glomerular and tublointerstitial samples. Since then, Nephromine has been updated into Nephroseq (www.nephroseq.org) and samples from the “Ju podocyte” dataset have been incorporated into the “Ju CKD Glom”, “Ju CKD TubInt” and “Ju CKD TubInt 2” datasets. Our search for Hsc70 expression was then conducted on the updated Nephroseq, using the Ju CKD Glom dataset, which is made up of micro-dissected glomeruli from 199 CKD patients or healthy living donors from the European renal cDNA Biobank (ERCB).

### 4.6. Immunofluorescence Staining of Cultured Podocytes and Quantification

Cells were plated on coverslips coated in laminin 521 (0.25 μg/cm^2^) and allowed to attach overnight. After treatment, cells were fixed in 4% paraformaldehyde for 15 min at room temperature, permeabilized with 0.5% Triton X-100 (in PBS), and blocked in 3% BSA (in PBS) for 20 min. Rabbit anti-Trio (1.5 h, 1:50), mouse anti-vinculin (1.5 h, 1:40), and rhodamine-conjugated phalloidin (20 min, 1:50) were used to stain Trio, vinculin, and filamentous actin, respectively. Samples stained for Trio and vinculin were then incubated with anti-rabbit IgG2 Fab2 Alexa Flour 488 and anti-mouse IgG Fab2 Alexa Flour 555, respectively, at 1:1000 dilution in 3% BSA for 1 h. Images were captured using the AxioObserver-100 microscope (Zeiss, Oberkochen, Germany). 

ImageJ was used to quantify cell size, focal adhesion complex (vinculin) number, and peripheral vinculin localization. Phalloidin staining was used to determine cell size. Particles between 1 and 8 μm^2^ in vinculin staining were quantified for focal adhesion complex number and then normalized to cell area, as described elsewhere [49]. For peripheral vinculin localization, the “Straight Line” tool was used in ImageJ to draw lines randomly across the cell and the fluorescence intensity was measured using a histograph. The fluorescence intensity in the outer 10 μm (i.e., the two ends of the line, touching the cell periphery) was the divided by the fluorescence intensity in the next 20 μm (i.e., from 10 to 30 μm from the periphery). 

### 4.7. Immunofluorescence Staining of Paraffin-Embedded Kidney Sections

Small pieces of a kidney were fixed in 100% methanol for 30 min at −20 °C, paraffin embedded, and sliced into 4 μm-thick sections. Slides were covered in xylene for 10 min two times. This was followed by a 10 min wash with ethanol, a 5 min wash with ethanol, and a 5 min wash with 95% ethanol (in PBS). They were then washed for 3 min in decreasing concentrations of ethanol (70%, 50%, and 30%) and finally in pure PBS for 5 min 3 times. Citrate antigen retrieval consisted of incubating slides in boiling 10 mM citrate buffer (pH 6.0) for 9 min. Slides were treated in 1% Triton X-100 (in PBS) for 30 min, and then blocked in 10% normal goat serum (NGS), 0.3% Triton X-100, and PBS for 1 h at room temperature. Incubation with first antibodies were overnight at 4 °C with rabbit anti-Trio, mouse anti-WT1, or mouse anti-podocalyxin antibodies (diluted 1:50 in 5% NGS, 0.3% Triton X-100 in PBS). Slides were washed 3 times (5 min) in PBS, and then incubated in secondary antibody (diluted 1:1000 in 0.3% Triton X-100 in PBS) at room temperature for 40–45 min. The secondary antibodies used were anti-rabbit IgG2 Fab2 Alexa Flour 488 and anti-mouse IgG Fab2 Alexa Flour 555. This was followed by three 10 min washes before mounting the coverslip with AquaMount.

### 4.8. SDS-PAGE and Immunoblotting

Cells were washed once with PBS and lysed with ice-cold lysis buffer (10 mM Tris (pH 7.5), 1 mM EDTA (pH 8.0), 1 mM EGTA (pH 8.0), 125 mM NaCl, 10 mM sodium pyrophosphate, 25 mM NaF, 2 mM sodium orthovanadate, 1% Triton X-100, and protease cocktail inhibitor cocktail). Lysates were briefly (4 s) sonicated at a low power and centrifuged (18,407 rcf, 30 s, 4 °C). The concentration of supernatant was determined by Bradford assay. Proteins were separated by SDS-PAGE, which were then transferred to nitrocellulose membranes.

Two systems of immunoblotting visualization were used: BioRad and LiCor. The membranes were handled differently depending on the visualization method. For the BioRad system, membranes were blocked with 5% skim milk (in Tris-buffered saline-Tween) at room temperature and then incubated in primary antibody overnight at 4 °C. The following day, membranes were washed twice (30 min each wash), incubated with secondary antibodies conjugated with horseradish peroxidase (dilution 1:1000–1:2000) for 1 h at room temperature. After two 30-min washes, proteins were visualized using ECL. When using the LiCor system, membranes were blocked for 1 h at room temperature, and then incubated in primary antibodies overnight at 4 °C. The membranes were then washed four times (5 min each wash), incubated with fluorescent secondary antibodies (dilution 1:20,000–1:10,000) for 1 h at room temperature, and then washed 4 times for 5 min again. After the addition of the secondary antibodies, the membranes were kept away from light. Proteins were visualized using the LiCor machine.

Densitometric analysis was performed using the BioRad or LiCor imaging systems. The following antibody dilutions were used: rabbit anti-Trio 1:100–1:1000; mouse anti-Rac1: 1:1000; mouse anti-p-p38 1:1000; mouse anti-p38 1:1000; rabbit anti-CdGAP 1:1000; rabbit anti-βPIX 1:1000; mouse anti-tubulin 1:5000; rabbit anti-calnexin 1:1000.

### 4.9. CRIB Pulldown Assay for Active Rac1

Active Rac1 was pulled down as previously described [46]. Briefly, the Cdc42-Rac1 Interactive Binding domain fused to GST beads (GST-CRIB) were coupled to glutathione-agarose beads. Cells were lysed on ice (as described above) and 100–150 μg was incubated with 15 μg GST-CRIB beads for 1 h at 4 °C. Pulldown samples were washed three times and proteins were separated by SDS-PAGE (12.5%). Densitometric analysis was performed using the BioRad imaging system. 

### 4.10. Trio and CdGAP Pulldown

Trio was pulled down using wild type Rac1 fused to GST. CdGAP was pulled down by constitutively-active Rac1 fused to GST. The GST-RacWT and GST-CA-Rac1 were all coupled to glutathione-agarose beads. Treated cells were lysed on ice, sonicated, and equal amounts of total cell lysates (550–1000 μg) were incubated with beads for 2 h at 4 °C. Pulldown samples were washed three times and separated by SDS-PAGE (5%). Densitometric analysis was performed using the LiCor imaging system. 

### 4.11. MTT Assay

For the MTT assay, 2 × 10^4^ cells/well were plated in a laminin 521-coated (0.25 μg/μm^2^) 96-well plate and incubated at 33 °C for 24 h to allow cells to attach. At 24 or 48 h, 3-(4,5-dimethylthiazol-2-yl)-2,5-diphenyltetrazolium bromide (MTT) was added to the medium to a final concentration 1 mg/mL. Cells were incubated for 4 h, washed and MTT uptaken by live cells were dissolved by acidified isopropanol (15 min, room temperature). The dissolved MTT was quantified using a microplate reader (550 nm, ELX808, Bio-Tek Instruments) with the KC Junior software (Bio-Tek, Winooski, VT, USA). Experiments were done in triplicates and repeated three times, and the proliferation rate was determined by normalizing the number of viable cells at 48 h to that at 24 h. 

### 4.12. Wound Healing Assay

Cells were plated on 35-mm plates, left to attach, and then serum starved overnight. The following day, the wound was created using a 10 μL pipette tip. Scraped off cells were washed off using PBS and then RPMI medium was replaced. Cells were visualized at 5× magnification using the AxioObserver-100 microscope (Zeiss) immediately after the wound was created and again after 5 h. Wound size was measured using ImageJ; wound size at 5 h was normalized to the original wound size and then wound closure rate of Trio KOs was normalized to that of control Cas9.

### 4.13. Attachment Assay

For the attachment assay, 10^4^ cells/well were added to a laminin 521-coated (0.25 μg/cm^2^) 96-well plate and allowed to attach for 1 h 15 min at 33 °C. Unadhered cells were washed away with PBS, and the adherent cells were fixed with 4% paraformaldehyde for 15 min. Fixed cells were washed and then incubated with 0.1% crystal violet dissolved in 200 mM 3-(*N*-morpholino) propanesulfonic acid (MOPS) for 15 min at room temperature. Cells were washed 3 times with PBS and then incubated for 15 min with 10% acetic acid. The dissolved crystal violet was measured using a microplate reader (550 nm, ELX808, Bio-Tek Instruments) with the KC Junior software (Bio-Tek). All experiments were done in triplicates and repeated 3–4 times.

### 4.14. Statistics

All data is presented as mean ± SEM. A two-tailed Student *t*-test is used to determine statistical differences between untreated and cells treated at each time point. 

## Supplementary Materials

Supplementary materials can be found at http://www.mdpi.com/1422-0067/19/2/479/s1.

## Abbreviations

CA	Constitutively Active
CKD	Chronic Kidney Disease
CRIB	Cdc42/Rac Interactive Binding
ECL	Enhanced Chemiluminescent
ERCB	European renal cDNA Biobank
FSGS	Focal Segmental Glomerulosclerosis
GST	Glutathione *S*-transferase
HRP	Horseradish peroxidase
KO	Knockout
LPS	Lipopolysaccharide
MAPK	Mitogen Active Protein Kinase
MOPS	200 mM 3-(*N*-morpholino) propanesulfonic acid
MTT	3-(4,5-dimethylthiazol-2-yl)-2,5-diphenyltetrazolium bromide
NGS	Normal Goat Serum
PBS	Phosphate Buffered Solution
PD	Pulldown
SDS-PAGE	Sodium dodecyl sulfate polyacrylamide gel electrophoresis
SEM	Standard Error of Mean
TGFβ1	Transforming growth factor β1
WT	Wild Type
WT1	Wilms Tumor 1
