# Supplementary material for: The Role of Trio, a Rho Guanine Nucleotide Exchange Factor, in Glomerular Podocytes
Source: Int J Mol Sci. 2018 Feb 6;19(2):479. doi: 10.3390/ijms19020479 (PMC5855701; doi:10.3390/ijms19020479)
Supplement: Supplementary file 1 [file ijms-19-00479-s001.pdf]

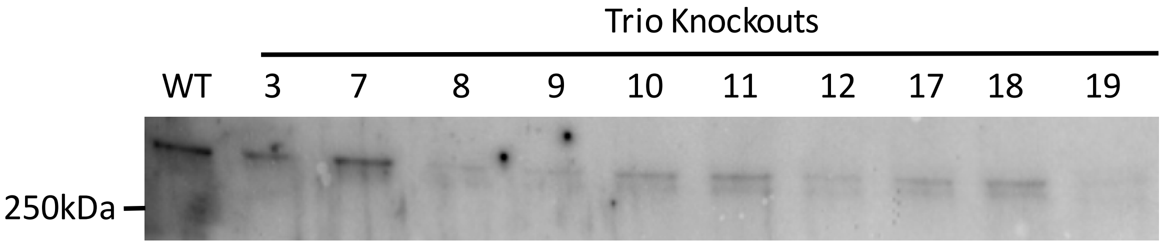

Figure S1. Expression of Trio in HP Trio KO Clones. Trio knockout clones 9 and 19 were used for proliferation studies.

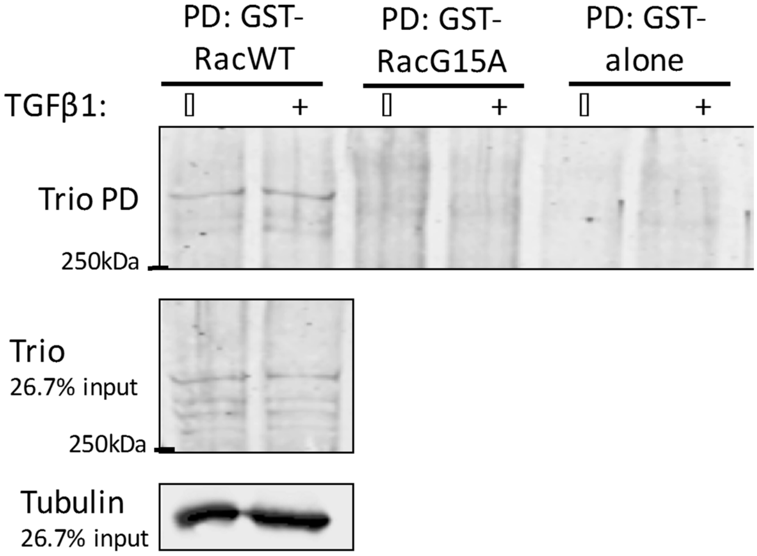

Figure S2. Trio is pulled down by GST-RacWT but not GST-RacG15A or GST-alone. MP were treated with TGFβ1 (60 min, 10 ng/mL) pulled down with GST-RacWT, GST-RacG15A, or GST-alone. Precipitates and lysates were blotted for Trio and tubulin. Three isoforms were pulled down by GST-RacWT (and increased with TGFβ1 stimulation), but no signal was found in the GST-RacG15A or GST-alone pull downs.

Table S1. RNA-seq Data of Trio-Regulating Proteins. Below are the FPKM values from our RNA seq data done on cultured LY cells (in duplicates, labelled (1) and (2)) and HP cells. We have analyzed the mRNA levels of proteins previously reported to control Trio's Rac1-GEF activity.

Protein	LY (1)	LY (2)	HP
Fyn	4.59	5.21	2.61
DISC1	0.29	0.34	0.64
Kidins220/ARMS	2.97	3.12	2.28
Tara	5.74	8.85	2.11
Hsc70	163.8	162.2	329.7
